# Concise set of files for smooth return to work in employees with mental disorders

**DOI:** 10.1186/2193-1801-2-630

**Published:** 2013-11-23

**Authors:** Kiyoshi Yoshitsugu, Yuko Kuroda, Yuji Hiroyama, Nobuhisa Nagano

**Affiliations:** Tokyo Marine and Nichido Medical Service Co. Ltd, Chiyoda, Tokyo, Japan

**Keywords:** Sick leave, Return to work, Workplace, General practitioner, Occupational physician

## Abstract

Sick leave due to mental disorders is a societal problem. It carries a high cost in terms of loss of labor productivity and absenteeism. Partial remission increases the risk of relapse after a return to work. There is sometimes a difference between the ability to return to work as judged by a general practitioner (GP) and the needs of the workplace. GPs are the main controllers of treatment and tend to protect their patients. Communication and agreement by GPs and occupational physicians play an effective role in the return to work. However, it requires considerable effort for both of them to make time to do this. We have developed a concise set of files for a smooth return to work. The files consist of three parts: “Suggestions for corresponding with employees taking sick leave”; “Checklist for smooth return to work”; and “Pattern of living”. We put them into practice among 20 companies in Japan from January 2012 to October 2013. The companies had 8244 workers in total and 116 workers were on sick-leave due to mental disorders. Our set of files contributed to sharing the written basic policy of return to work among employees on sick leave with mental disorders, GPs, occupational physicians and personnel officers. That sharing led to facilitating a smooth return to work. Although there are differences in the legal and medical systems between Japan and other countries, our concept of sharing the written basic policy may give some help to occupational physicians in other parts of the world as well.

## Background

Mental disorders (such as depression, bipolar disorder, developmental disorder, personality disorder, anxiety disorder, and adjustment disorder) have negative effects on workers. They are a major cause of disability in many Western countries and Japan (Dekkers-Sánchez et al. 2008;Henderson et al. 2005;Kawakami et al. 2005;Sado et al. 2011). Mental disorders result in long-term absenteeism. Only 50% of those workers who are classified as sick for ≥6 months return to work, which causes a large loss of workforce in society (Blank et al. 2008;Brouwers et al. 2006a). This may also result in financial loss for each worker.

The Netherlands has a national system of care for employees who suffer from serious mental disorders, take long sickness leaves, and try to return to work (van der Klink & van Dijk 2003a;van der Klink et al. 2003b). In contrast, Japan has no national system to give support to such people. The Ministry of Health, Labour and Welfare of Japan (MHLW) published guidelines for sick-listed employees with mental disorders returning to work (Ministry of Health labor and Welfare of Japan 2009). Returning to work requires an intensive approach for all the people concerned.

Generally, a medical certificate by a general practitioner (GP) is not in itself sufficient to enable a return to work. The first duty of GPs is to support their patients as much as possible. However, they have many patients to see every day and they cannot assign much time to all of them. If patients who are unable to return to work explain that they will lose their job, GPs might fill out a medical certificate saying that they are able to work. Some GPs state that patients can return to work under the condition that their hours are limited to 3 hours daily for 6 months. Taira (2012) commented that their attitudes were too prudent. Pintor et al. (2003) reported that partial remission after a depressive episode seems to be strongly associated with relapse. They asserted the importance of achieving complete remission to decrease the rate of short-term relapse. Antidepressant treatment is not a panacea for mental disorders, and may leave patients with some residual symptoms (McClintock et al. 2011). Hence, overprotective judgment toward patients by a GP may lead to relapses after return to work. It also may lead to loss of morale among employees and problems for managers.

Occupational physicians are expected to hold a neutral position between their patients and the companies for whom they work. The MHLW guidelines require occupational physicians to play an active and important role in return to work support. Additionally, it is sometimes difficult for a GP to gain access to a company. Occupational physicians have the opportunity to make contact with the workplace and adjust the environment. Previous studies indicate that intervention by occupational physicians reduces sick leave duration (Nieuwenhuijsen et al. 2004;Anema et al. 2006;Brouwers et al. 2006b).

In this paper, we describe how we presented a new method that occupational physicians could use to deal with return to work in employees with mental disorders and then assessed its effects.

## Results

### Participating companies

We performed our study only in Japan. Most of the companies were situated in the Kanto region of Japan, centered around Tokyo. We proposed our set of files to 20 companies and then they adopted it. Their size of business varied from small to large. The smallest company had 56 employees and the largest company had 1417 employees. The companies had 8244 workers in total and 116 workers who were on sick-leave due to mental disorders. They conducted business in various sectors, for example, finance, real estate, and the leasing industry. This study was conducted from January 2012 to October 2013.

### Employees’ diagnosis

Employees beginning sick leave for mental disorders submitted medical certificates from their GPs. Experienced occupational physicians majoring in occupational mental health judged whether each employee actually had a mental disorder, according to that certificate and all the relevant medical information. Our main reference diagnostic criteria were from the Structured Interview for the Diagnostic and Statistical Manual of Mental Disorders, Fourth Edition Text Revision (DSM-IV-TR) Axis I Disorders (American Psychiatric Association 2000).

### Supplementary rehabilitation program

In principle, the “Rework Program” in Japan was introduced to employees who took sick leave for mental disorders more than three times. It was also introduced to employees with developmental disorders. The consideration of whether an employee had a developmental disorder or not was primarily based on a medical certificate by his GP. In addition, when both an occupational physician and personnel officers suspected that an employee had a developmental disorder, obtaining the opinions of his GP was actively sought with his permission. In addition to these two types of employees, some employees attended the “Rework Program” voluntarily by way of consulting their GPs.

### Criteria for return to work

As the MHLW described, it is difficult to show fixed criteria for return to work and it is necessary to judge comprehensively in accordance with each case (Ministry of Health labor and Welfare of Japan 2009).

We demanded that an employee accomplish the eight points by the MHLW referred to in Method section of this paper at a minimum. We utilized the “Checklist for smooth return to work” and the “Pattern of living”. For example, it is too early to return to work if the employee has a habit of lying in bed late in the morning or staying up late at night; if the employee requires to take a nap for 3 hours during the day; or if the employee cannot concentrate on reading work-related news articles for a fixed period of time.

We also demanded that he analyze factors that caused or affected his worsening condition and take preventive measures. For example, if he tends to accomplish everything at work by himself, it would be better for him to ask his colleagues for assistance.

For the employees attending the “Rework Program”, we could receive reports about them from their institutions regularly and take the reports into consideration. For example, if a report asks for some special care in communication with an employee with a developmental disorder at his company, the company examines it as much as possible.

### Qualitative results

The written basic policy of return to work was shared among employees on sick-leave due to mental disorders, GPs, occupational physicians and personnel officers. Information sharing through documents had not occurred prior to the introduction of our set of files.

**Figure 1:**
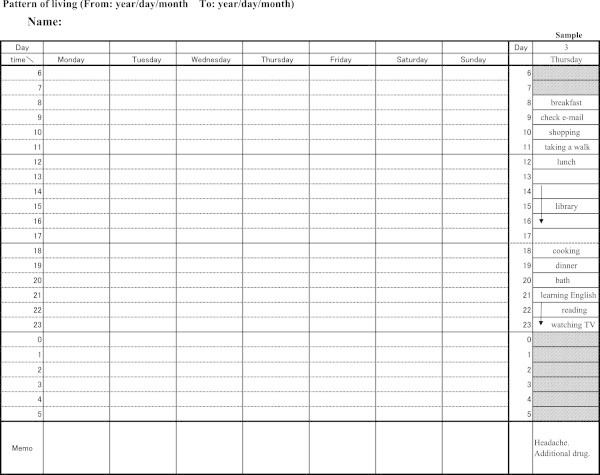
**Suggestions for corresponding with employees taking sick leave.**

This program took effect as follows: 1) Before applying for return to work, an employee took medical treatment considering the “Checklist for smooth return to work” (see the Methods section) and the “Pattern of living” (Figure 1). We did not observe opposite opinions or objections against the “Checklist for smooth return to work” or the “Pattern of living” either from employees or GPs. Personnel officers contacted the employee considering the “Suggestions for corresponding with employees taking sick leave” (see the Methods section) and gave attention to when he would apply for return to work; 2) When an employee applied for return to work, his occupational physician and personnel officers could assume that he had understood and practiced the “Checklist for smooth return to work” and the “Pattern of living” and dealt with the issues outlined there; 3) After introducing an employee to the process of returning to work, personnel officers treated him according to the “Suggestions for corresponding with employees taking sick leave”.

## Discussion

We connected employees on sick-leave due to mental disorder, GPs, occupational physicians and personnel officers with our concise set of files.

It is not easy for occupational physicians to manage the situation of returning to work. First, the main controllers of treatment during sickness leave are GPs. Their role is to support their patients as much as possible to provide them with sufficient recovery. GPs can prescribe medication and perform continuous psychotherapy. In contrast, occupational physicians can do neither of these things. Consequently, they are hesitant to approach employees during sick leave. Second, companies are prohibited by Japanese law to give business orders to employees who are on sick leave. The company can recommend that workers consult with an occupational physician, but they cannot force them to do that. If the employment practice at a particular company has regulations relating to this, there might be room to encourage employees to consult an occupational physician. Even if this consultation occurs, it carries the risk of making matters worse. Third, employees sometimes think that occupational physicians favor the company. That is because the company pays the salary of the occupational physicians in Japan, although the physician has a neutral position between the employee and the company. The results of this study suggest that information sharing through documents reduces the burden of occupational physicians.

In Japan, some hospitals, clinics and institutions have rehabilitation programs for returning to work. They are commonly called “Rework Programs” (Akiyama et al. 2010). Such programs involve outpatient occupational therapy. Regular and routine attendance assists with the recovery of physical strength. Participants may have the opportunity to strengthen their cognitive function, and learn skills to cope with stress and cooperate with other members of staff. Some research teams have reported that cognitive behavioral intervention is effective for returning to work (van der Klink et al. 2001;Blonk et al. 2006;Lagerveld et al. 2012). The best method to achieve a smooth return to work might be evaluation of recovery by those with specialized knowledge. Not all employees on sick leave can take part in these return to work programs. They need to be working for institutions that have adequate space, staff and money. Completing the programs often demands time on a monthly basis. In principle, we introduced the “Rework Program” to the employees who took sick leave for mental disorders more than three times. That was because they could not succeed in returning to work through approaches with insufficient time and labor, for example, guidance to improve daily living only by their GPs. Moreover, judicial precedents could support this introduction to employees who took sick leave more than three times. We also introduced the “Rework Program” to employees with developmental disorders. People with developmental disorders have some specific characteristics, such as impairment of interpersonal communication (Wing & Gould 1979). Consequently, they have difficulty maintaining their jobs (Hurlbutt & Chalmers 2004). It is important for employees with developmental disorders to receive careful training on interpersonal communication and to have coordination with their working environment. The people involved in our cases collaborated with each other, but this cannot be assumed.

Some employees on sick leave undertake private training or rehabilitation to assist their return to work. If they do not follow definite guidelines, their program may have insufficient content; for example, Web surfing and reading discursively. Most work involves time limits and checking by managers. Our files support connecting daily life in sick leave with activities in the workplace.

Researchers on returning to work in the Netherlands proposed protocols and carried out several trials (Rebergen et al. 2007;van Dijk et al. 2008;Vlasveld et al. 2012;van Beurden et al. 2013). Japan has its own legal and medical system different from other countries including the Netherlands, but it would be a common challenge for all to improve the smooth return to work in employees with mental disorders. Our concept of sharing the written basic policy may give some help to occupational physicians in foreign countries.

The limitation of this study exists in its qualitative assessment. Our method arranged the complicated process of returning to work for employees with mental disorders and led to the facilitation of a smooth return to work. We were able to show this in a qualitative way. Accordingly, quantitative studies need to be performed to confirm our findings in the future, such as changes in the success ratio of return to work.

## Conclusions

We presented a new method that occupational physicians could use to deal with return to work of employees with mental disorders. Our method made it possible to share the written basic policy of returning to work among employees, GPs, occupational physicians and personnel officers. That sharing led to facilitating a smooth return to work. Although there are differences in legal and medical systems between Japan and other countries, our concept of sharing written basic policy may give some help to occupational physicians in other parts of the world.

## Methods

### Components of our set of files

We conceptualized and developed a concise set of files for a smooth return to work for employees with mental disorders. The files consist of three parts: “Suggestions for corresponding with employees taking sick leave”; “Checklist for smooth return to work”; and “Pattern of living” (Figure 1).

***Suggestions for corresponding with employees taking sick leave***

### **How to contact them**

An immediate superior or a person in charge of personnel management handles the contact. Establish that they will report their living conditions and hospital attendance approximately every other week (or once a month if the mental burden is too large).

Ask them to choose the method of communication with least burden, such as e-mail or telephone.

**Matters that would be better made known early in the leave period**

1. Leave expiration date
2. Criteria for return to work
  Example:
  - The employee is able to complete standard work and work regular office hours.
    (Note: You will not permit a return to work if all an employee can do is part-time work)
  - You can confirm the employee’s positive willingness to perform his/her duties.
  - The employee can commute unaided and safely during the required hours.
  - The employee can perform work, such as reading or computer operations, and has recovered adequate attention and concentration.
  - You can confirm that the employee has recovered almost the same regular pattern of living as ordinary workers, through the sheet “Pattern of living”.
3. How to treat after return to work (It is preferable to make unified rules in the company if possible.)
  Example:
  - We will not require overtime or holiday work for one month after the return to work.
    (Note:Even if it is difficult to impose restrictions, we advise you to set regular work hours for at least one month from the point of view of safety obligations)
  - Part-time work may be permitted for at most one month based on the opinion of the occupational physician. The employees covered are those who can complete standard work, and work regular office hours.
    (Note: The execution of part-time work is not a legal obligation of companies, so it is all right even if your company will not permit it. In general, its period is two to four weeks at the longest. If an employee requires part-time work for more than four weeks, you had better not permit his return to work.)
  - We may restrict engagement, such as limit overtime, or limit employment for up to three months, based on the opinion of the occupational physician.
    (Note: Some companies have a principle of three to six months)

We recommend that you notify the employees through documentation and urge them to share these guidelines with their GPs. These are important to identify criteria for the return to work and establish the limit of restrictions on engagement. The company should try to help as much as possible but it is impossible to treat employees in an exceptional way without restriction.

**Interview just before expiration of sick leave**

Conflicts may occur with regard to the return to work judgment just before expiration of the sick leave period. That is because it is directly related to employment issues. We would ask you to settle the return to work judgment at least 1 month before sick leave expiration.

Therefore, we would ask you to consider the following.

1. At least two months before the expiration date, make contact with us and make a tentative reservation for an interview.
  After the reservation, give the employee the “Checklist for smooth return to work” and “Pattern of living”.
2. Ask the employee to submit a medical certificate from a GP in time for the interview held one month before expiration of sick leave.

**What the employee brings on the day of interview by the occupational physician**

1. Checklist for smooth return-to-work
2. Pattern of living

* With respect to “Checklist for smooth return-to-work”

Employees on sick leave begin to think about a return to work soon after subjective symptom improvement. Some of them apply for a return to work without any actual preparations, for instance, even if they have a habit of lying in bed late in the morning or staying up late at night, or do not leave home. Such cases of panic generally result in failure to return to work. We have prepared a set of files that promotes a return to work with adequate medical treatment. Ask the employees to fulfill the items on the list, and then arrange for an interview with an occupational physician after they have applied for a return to work. That should lead to a smooth return to work. It is better to give the set of files to the employee early in the sick leave period.

***Checklist for smooth return to work***

If there is a gradual improvement in symptoms, then you may think of a return to work. Many employees on sick leave spend most of their time at home, so they have much less activity compared to that of daily commuting. Therefore, for your smooth return to work, it is important during sick leave at home to undergo rehabilitation assuming that you are at work.

We recommend that you take adequate rest and establish a proper pattern of living. With regard to starting the time of rehabilitation, it is not advisable to be impatient. We would also recommend that you consult your GP and make a reasonable plan.

The following checklist includes the items that we ask you to practice. We hope that you can apply for a return to work after checking whether you can easily carry out these items.

- □ Can you go to bed and get up at a period of time suitable for commuting?
  (Note: If you have a habit of lying in bed late in the morning or staying up late at night, you might not resolve this disturbance of your sleep pattern even after a return to work and might have trouble going to work)
- □ Can you stay awake during the day without a nap?
  (Note: You cannot have a nap at your own volition after you return to work; therefore, you need to check whether drowsiness arises during the day)
- □ Can you do moderate exercise such as walking?
- □ Can you go out with your current appearance?
- □ Can you spend time in public places such as libraries?
- □ Do you have a practice of commuting?
  (Note: It is particularly tiring to commute in the morning rush hours. We recommend that you practice and become used to commuting before your return to work on the condition that you consult your GP, for example, going to the vicinity of your company around the actual commuting time for at least 1–2 weeks.)
- □ Can you perform computer operations such as simple data input, producing graphics, or checking the internet associated with your work?
  (Note: This is for employees who work with personal computers)
- □ Can you intensively read newspaper articles, magazines and books related to your work?
  (Note: It is effective to report your favorite story or summarize the contents of a book within a limit of 30–60 minutes.)

* At an interview for judging your return to work, we would ask you to record and bring the attached sheet “Pattern of living”. It is desirable that the period of recording is more than 2 weeks before the interview.

### Basis of our set of files

Occupational health in Japan is covered by national laws and the guidelines of the MHLW. Some of these laws are: Labor Standards Act, Industrial Safety and Health Act, and Labor Contract Act. Japanese companies set their own rules for employment on the basis of these laws. Obligations considering safety are so important that Japanese companies must prohibit the continued employment of sick people. Taking into account judicial precedents, recovery is required for employees on sick leave for mental disorders to return to work. In practical terms, recovery means being able to accomplish regular work duties for each employee.

The MHLW has published guidelines for sick-listed employees with mental disorders returning to work. For instance, one type of criteria for return to work was quoted and it required the following eight points: 1) An employee is sufficiently willing to return to work; 2) He can commute safely by himself during commuting hours; 3) He can continue to work on duty for the work days that his company has set; 4) He can carry out operations necessary for his work, for example, reading, computer-operation and light exercise; 5) He can sufficiently recover from his exhaustion caused by work by the next day; 6) He has retained adequate sleep-awake rhythm; 7) He has no daytime sleepiness; 8) He has recovered attention and concentration necessary for his work.

We developed our set of files based on the knowledge described above.

### Procedure

An occupational physician contracted by the company holds a meeting with the person in charge of the employee’s mental disorder as soon as possible. The aim is to share and deepen their understanding of our set of files. This enables the person in charge of the employee’s mental disorder to learn a basic framework of thinking about the return to work.

When an employee takes sick leave for a mental disorder, the person in charge gives the employee the “Checklist for smooth return to work” and the “Pattern of living.” It is better to give these to the employee early in the time of the sick leave. The employee then takes the files to a GP. If the employee or GP has any questions about the files, the occupational physician will be able to answer them.

During medical treatment, all those involved – employees on sick leave, GPs, occupational physicians, and the personnel division – check and assess the current status while referring to the file set. It is especially important to confirm accomplishment of the items when a GP provides a medical certificate of return to work.

In accordance with our system, the occupational physician sets an interview with the employee to judge the readiness to return to work. The occupational physician carefully verifies the present health status of the employee and reports to the company.

Eventually the personnel department decides whether it will accept the application for return to work, considering all the conditions included in our set of files.
